# ﻿Amplicon metagenomics of dung beetles (Coleoptera, Scarabaeidae, Scarabaeinae) as a proxy for lemur (Primates, Lemuroidea) studies in Madagascar

**DOI:** 10.3897/zookeys.1181.107496

**Published:** 2023-09-28

**Authors:** Andrey V. Frolov, Lilia A. Akhmetova, Maria S. Vishnevskaya, Bogdan A. Kiriukhin, Olivier Montreuil, Fernando Lopes, Sergei I. Tarasov

**Affiliations:** 1 Zoological Institute, Russian Academy of Sciences, Saint Petersburg, Russia Zoological Institute, Russian Academy of Sciences Saint Petersburg Russia; 2 Department of Entomology, Saint Petersburg State University, Saint Petersburg, Russia Saint Petersburg State University Saint Petersburg Russia; 3 AquaBioSafe Laboratory, University of Tyumen, Tyumen, Russia University of Tyumen Tyumen Russia; 4 National Museum of Natural History, Paris, France National Museum of Natural History Paris France; 5 Finnish Museum of Natural History, University of Helsinki, Helsinki, Finland University of Helsinki Helsinki Finland

**Keywords:** Beetles, coprophagy, gut content analysis, Madagascar, next-generation sequencing, scarabaeines

## Abstract

Dung beetles (Scarabaeidae, Scarabaeinae) are among the most cost-effective and informative biodiversity indicator groups, conveying rich information about the status of habitats and faunas of an area. Yet their use for monitoring the mammal species, that are the main providers of the food for the dung beetles, has only recently been recognized. In the present work, we studied the diet of four endemic Madagascan dung beetles (*Helictopleurusfissicollis* (Fairmaire), *H.giganteus* (Harold), *Nanosagaboides* (Boucomont), and *Epilissussplendidus* Fairmaire) using high-throughput sequencing and amplicon metagenomics. For all beetle species, the ⅔–¾ of reads belonged to humans, suggesting that human feces are the main source of food for the beetles in the examined areas. The second most abundant were the reads of the cattle (*Bostaurus* Linnaeus). We also found lower but significant number of reads of six lemur species belonging to three genera. Our sampling localities agree well with the known ranges of these lemur species. The amplicon metagenomics method proved a promising tool for the lemur inventories in Madagascar.

## ﻿Introduction

Madagascar is known for its unique biota characterized by an exceptionally high level of endemism at all taxonomic levels (Vences et al. 2009). Species-level endemism reaches 100% in many taxa, and there are many families and tribes that are found only in Madagascar. For example, all native amphibians and land mammals in Madagascar are endemic (Goodman and Benstead 2003). Due to its exceptional endemism and generally high species diversity, Madagascar is considered one of the most important biodiversity hotspots in the world (Myers et al. 2000), yet its biodiversity is highly underestimated (Vieites et al. 2009).

Dung beetles are a marquee focal group for global efforts to assess the status of biodiversity. They are among the most cost-effective and informative biodiversity indicator groups (Spector 2006; Nichols and Gardner 2011). Dung beetles provide rich information about the status of habitats and faunas of an area (Davis et al. 2004; Nichols et al. 2008; Scholtz et al. 2009). In Madagascar, they are important elements in forest food chains and ecosystems where they originally evolved to decompose lemur excrements (Orsini et al. 2007; Wirta and Montreuil 2008; Wirta et al. 2008; Viljanen et al. 2010; Wirta et al. 2010; Montreuil and Viljanen 2022). Increasing anthropogenic pressure reduces forest habitats, where bulk of dung beetles occurs, and population of lemurs, the original producers of food for dung beetles. This forces dung beetles to switch to other food sources including human feces and cattle dung. This drastically affects distribution, population size, and survival of dung beetles, and leads to a global rewiring in tropical food chains (Hanski et al. 2008; Rahagalala et al. 2009; Wirta et al. 2014).

Recently, attempts have been made to use DNA metabarcoding with High-Throughput, or Next-Generation, sequencing (NGS) to identify taxa in material samples, such as plant species in herbivore dung, prey species in the gut contents of predators, or soil samples (Taberlet et al. 2012; Kocher et al. 2017). Animal manure includes not only the DNA of the food, but also the DNA of the animal producing the manure (Shehzad et al. 2012; De Barba et al. 2014; Lopes et al. 2023). Three studies have been published using NGS to identify mammalian food sources for coprophagous beetles. Gillett et al. (2016) tested the intestinal contents of 10 specimens of different species of coprophagous beetles collected using window traps in the Mbuluzi Game Reserve, eastern Swaziland. In these samples, they found DNA from seven species of mammals, including two species of cattle, domestic mice, and humans. Kerley et al. (2018) used the flightless beetle *Circelliumbacchus* (Fabricius) to study changes in its diet, identify beetle food sources using DNA metabarcoding, and compare with published larval feeding data. Traditionally, *C.bacchus* was thought to specialize in the dung of large herbivores as a food source for both larvae and adult beetles. Kerley et al. (2018) extracted mammalian DNA from 151 adult *C.bacchus* fecal samples and sequenced them from 16 mammalian species (ranging from elephants to small rodents), many of which were not yet known as a food source for this species. This approach also confirmed the presence of unknown taxa of mammals in the study area. These authors suggested that the data obtained could be used to study and monitor the biodiversity of mammals. Mouse-like rodent feces were the most common food source (77.5%) for adult *C.bacchus*, which differed markedly from dung-bearing large herbivores used to raise larvae. These results support the hypothesis of changes in the use of food resources in dung beetles associated with the life cycle and reveal a previously unknown but ecologically important role of these beetles in the disposal of rodent feces. Drinkwater et al. (2021) studied gut content of large dung-beetle species and communities of smaller beetles in lowland tropical rainforest in Sabah, Malaysia. They successfully identified six mammalian species, including bearded pig (*Susbarbatus* Müller) and sambar deer (*Rusaunicolor* (Kerr)).

The main goal of the present study was to evaluate the amplicon metagenomics methods to find out what are the major food providers for a few model Madagascan dung-beetle species. We did not aim at studying the complete diet of these species, nor did we study differences in the diet among individuals or groups. Instead, the study was designed to include specimens of different taxa collected in a few different areas where both native and introduced animals are available as potential food producers for dung beetles. We were specifically interested in finding sequences of lemurs (Lemuroidea) because of their importance for nature conservation in Madagascar. For preparation of amplicon libraries, we used the primers for the 16S rRNA gene fragment, successfully utilized by Ji et al. (2022) to study trophic associations of leeches. Another primer pair, used by Ji et al. to amplify 12S rRNA gene fragment, was tested but not adopted, because, being highly degenerate, it is less specific for mammalian taxa and is not species-specific for some lemur species.

## ﻿Materials and methods

### ﻿Materials and collection methods

**Figure 1.**
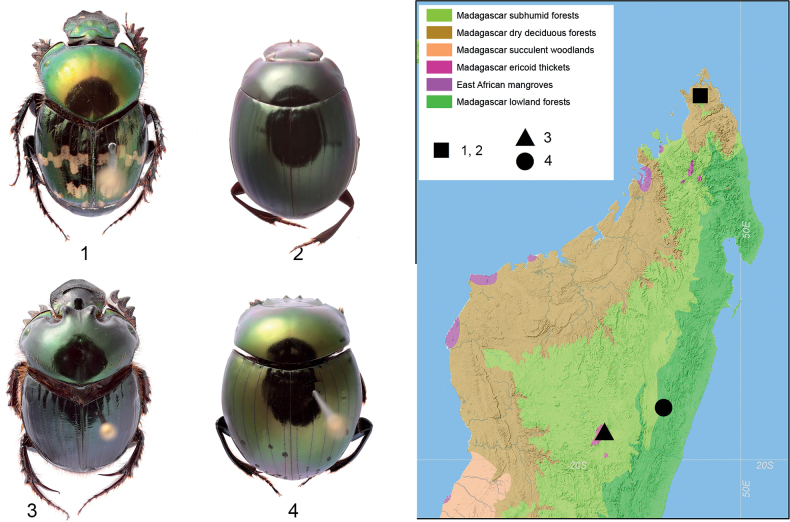
Madagascan Scarabaeidae dung beetles, general view of beetles (left, not to scale) and sampling localities map (right) **1***Helictopleurusfissicollis* (Fairmaire) **2***Nanosagaboides***3***H.giganteus* (Harold) **4***Epilissussplendidus* Fairmaire.

Examined material is housed in the collection of Muséum national d’Histoire naturelle, Paris (**MNHN**), and Zoological Institute, Saint Petersburg, Russia (**ZIN**). Beetles were collected in four localities in northern (Montagne d’Ambre National Park and Montagne d’Ambre Nature Reserve) and central (Ankaratra Andraraty Forest Reserve and Andasibe-Mantadia National Park) Madagascar (Fig. 1):

*Helictopleurusfissicollis* (Fairmaire). Madagascar • 12 females and 5 males (ZIN), Montagne d’Ambre National Park, 12°31'29″S, 49°10'19″E, forest, 5–10.02.2022, A.V. Frolov leg.

*Helictopleurusgiganteus* (Harold). Madagascar • 5 females and 4 males (ZIN), Ankaratra Andraraty, 19°21'20″S, 47°18'18″E PFT, cow dung 23.02.2022, A.V.Frolov leg.

*Nanosagaboides* Madagascar • 12 females and 6 males (MNHN), Montagne d’Ambre Nature Reserve, 12°28'15″S, 49°13'4″E, forest, 7–10.02.2022, A.V. Frolov leg.

*Epilissussplendidus* Fairmaire. Madagascar • 2 females and 4 males (MNHN), Andasibe-Mantadia National Park, 18°49'31″S, 48°26'5″E, 18–20.II.2022, A.V. Frolov leg.

The beetles we collected by standard pitfall traps baited with human feces (all except *H.giganteus*) and from cow dung pads (*H.giganteus*). The trap captured *H.fissicollis* contained a preservation solution with SDS and EDTA (Pokluda et al. 2014) and was exposed for 5 days until picking up. In other pitfall traps, we used funnels over the collecting jars, so the beetles attracted to the traps fell into the jars and stayed alive until picking up; these traps were exposed overnight. After picking up, the beetles were placed in containers with 96% ethanol and transported to the laboratory in two or three weeks at room temperature; the alcohol was changed the next day after collecting and after a week. At the laboratory, the beetles were placed in a freezer until dissection.

### ﻿Gut content extraction

For the analysis, the preserved beetles were dissected under a stereomicroscope. Abdominal tergites were cut with micro-scissors and, if the gut had visible content, it was dissected and placed in Eppendorf micro-tube with 96% ethanol for DNA extraction. Gut content was extracted from eight specimens.

### ﻿DNA extraction and sequencing

DNA was extracted by phenol-chloroform extraction method (Sambrook and Russell 2006) [*H.giganteus*] or using a diaGene extraction kit (Dia-M, Moscow) according to the manufacturer’s protocol. The extracted DNA was quantified using a Qubit fluorimeter 4.0 with high-sensitivity reagents (Lumiprobe QuDye dsDNA HS Assay Kit) and 1 µl of DNA. Four samples from different species with highest concentration of the extracted DNA were selected for high throughput sequencing. For amplicon metagenomics sequencing with the following primer pairs were used: 16Smam1 (5'-CGGTTGGGGTGACCTCGGA-3') and 16Smam2 (5'-GCTGTTATCCCTAGGGTAACT-3') (Taylor 1996). Libraries were prepared using the NEBNext Ultra II DNA Library Prep Kit, checked with Qubit (high-sensitive reagents) and real-time PCR for quantification, and Bioanalyzer for size distribution detection. The amplicon paired-end libraries (PE250) targeting an insert size of 350 bp were sequenced on Illumina NovaSeq 6000 platform aiming for 30K raw tags per sample. DNA extraction was performed at “Chromas” Core Facility, Saint Petersburg State University (Peterhoff, Russia), and library preparation, quality control, and sequencing were performed at Novogene (Novogene, Cambridge, UK). The data presented in the study are deposited in the NCBI Sequence Read Archive (SRA) database, accession number PRJNA958125.

### ﻿Bioinformatics methods

Raw reads were trimmed from adapter reads using bbduk. CutAdapt 4.3 was used to trim primer reads and to discard reads without primers (Martin 2011). Quality filtering was applied with the Dada2 (Callahan et al. 2016) command «filterAndTrim» with total expected errors = 1 for forward reads and 2 for reverse reads. For reads visualization and quality checking FastQC tool was used on each of previous steps. Standard Dada2 pipeline was used for further ASV producing. Taxonomy assignment was carried out using the BLASTN (McGinnis and Madden 2004) algorithm using the NCBI nucleotide database as a reference. ASVs with fewer than 10 copies were removed to minimize impact of likely spurious reads. The number of reads of each sample was rarefied by the value of the sample with the smallest number using the phyloseq (McMurdie and Holmes 2013) function «rarefy_even_depth».

To validate the identification of the hosts, we recovered a maximum-likelihood (ML) phylogeny with the most representative ASVs from each sample and reads obtained from GenBank for closely related species under the same genera. Reads were aligned, manually trimmed, and the ML phylogeny was recovered with MEGAX (Kumar et al. 2018).

## ﻿Results

We have successfully amplified 16S rDNA marker from all samples with a total 363558 reads; 326522 reads passed through quality filtering and preprocessing procedures. Filtered reads were denoised and formed 150 ASVs. Taxonomy assignments produced by BLASTN were manually checked to address possible “overclassification” due to similarity of the marker (for example in Hominidae species). The results are summarized in Table 1.

**Table 1.** Results of the amplicon metagenomic analysis of the gut content of four Madagascan Scarabaeidae dung beetles species.

Mammal species	*Nanosagaboides*	*Helictopleurusfissicollis*	*Epilissussplendidus*	*Helictopleurusgiganteus*
*Homosapiens*	55074	44855	80293	56315
*Eulemurcoronatus*	0	329	0	0
*Eulemurfulvus*	0	0	28	63
*Eulemurrubriventer*	9	13	1161	0
*Eulemursanfordi*	2853	14803	0	0
*Hapalemurgriseus*	0	0	212	0
*Propithecusdiadema*	3	6	425	0
*Bostaurus*	13224	3895	12481	28376
*Bovidae*	0	166	0	1
*Canislupus*	0	93	0	2
*Susscrofa*	256	700	323	427
*Arvicolinae*	912	0	0	4
*Ellobiustalpinus*	427	223	1180	6
*Lemmuslemmus*	8	7	0	3907
*Rattusnorvegicus*	412	189	1	2860

The total number of mammalian taxa identified includes 15 species with most of them belonging to primates (7 species including human). Small number of reads was classified as belonging to domestic animals (dog), and may also be considered contaminations, although dogs may occur in the studied areas. The *Nanosagaboides* sample yielded reads of Arvicolinae rodents, not occurring in Madagascar. The *H.fissicollis* sample yielded reads of Bovidae, having high but not 100% similarity with a number of ruminant taxa. The *H.giganteus* sample yielded reads of Norway lemming (*Lemmuslemmus* (Linnaeus)), which does not occur in Madagascar and is apparently a contamination (this sample was processed separately in the DNA extraction laboratory). All but *H.giganteus* samples yielded reads of northern mole vole (*Ellobiustalpinus* (Pallas)). We consider these reads as contaminations, because these animals do not occur in Madagascar but they were studied in the past years in the molecular laboratory where the DNA was extracted.

**Figure 2.**
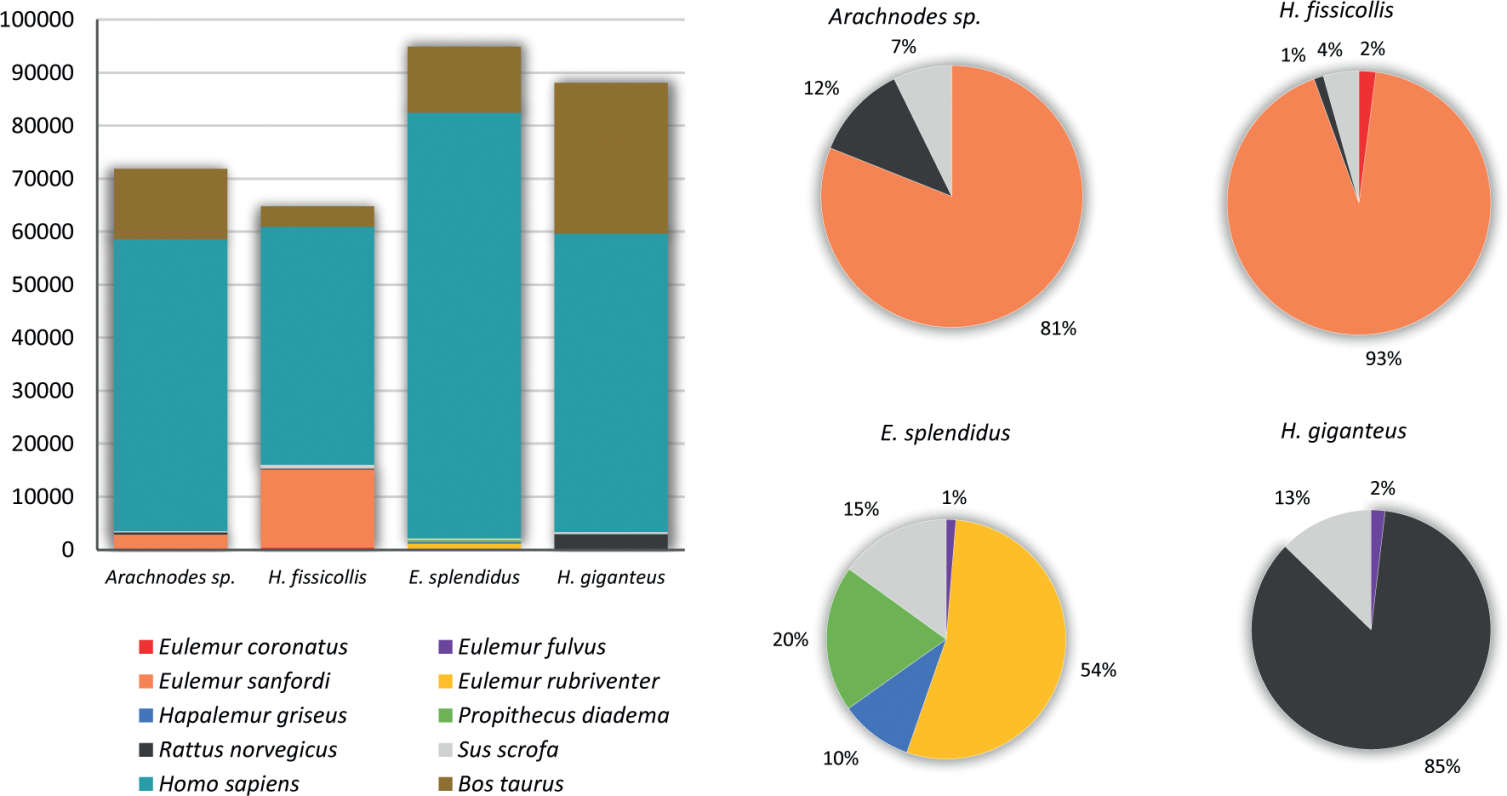
Results of the amplicon metagenomic analysis of the gut content of four Madagascan Scarabaeidae dung-beetle species (contamination reads are excluded). Left, number of effective tags per beetle sample. Right, proportion of effective tags per beetle sample, excluding human and cattle sequences.

The mammal species that we think were true sources of the food for the examined beetle species (Fig. 2) are discussed below.

## ﻿Discussion

The major challenge in studying dung-beetle gut content by molecular methods is that the DNA of the putative food producers is highly degraded and is generally in low quantity. At the same time, the total DNA is extracted from the samples of the gross guts dissected from the beetles, and it is hardly possible with the beetles of this size to separate gut content from the gut tissue. In addition, the gut content is normally full of symbiotic microorganisms. Therefore, using shotgun NGS sequencing for this DNA will be economically and computationally suboptimal since the great majority of the reads would belong to non-target organisms.

Our results demonstrated that instead of shotgun approach, the amplicon metagenomic method could be successfully applied to investigate the diet of Madagascan dung beetles and the composition of the local communities of animals that provide food resources to them.

It should be noted that in all samples 2/3–4/5 of the reads belong to humans. Part of the reads might result from the contamination by the bait, although the traps were designed in such a way as to minimize possible contact of the beetles with bait. Even if the beetles manage to consume bait, it may contribute to the total reads but not substitute the reads from the dung consumed before. In addition, since the beetles are fixed immediately or shortly after they arrived to traps, the foodstuff from the bait may not go through digestive system to get to the hindgut. The contamination is also possible during the laboratory work. However, we believe the human contaminations can be small or negligible percent of all reads we encountered.

The second most abundant reads belong to cattle (*Bostaurus* Linnaeus). Not surprisingly, they are the most abundant in the *H.giganteus* sample, where the beetle was collected from a cattle dung pad. But a reasonable amount of reads were also found in the *H.fissicollis* sample from Amber Mountain National Park, collected by pitfall traps a few kilometers inside the park.

A high number of reads of humans (more than 2/3 in our analyses) and cattle, which was similar for different samples, regardless of the taxonomy of the beetles and collecting localities, shows that humans and cattle can be considered as the main food producers at least for some native Madagascar dung beetle taxa.

In all our samples (except for *Epilissussplendidus*) we revealed the sequences of Norway rat (*Rattusnorvegicus* (Berkenhout)). However, with the data available we cannot be sure that in all these cases rats were the real food producers for the beetles. It is possible that in case of *H.giganteus* at least a part of sequences might be a result of contamination in the laboratory, which previously processed rodent samples. *Helictopleurusgiganteus* sample also yielded reasonable number of lemming reads, which are definitely a result of contamination.

However, the revealed sequences of our main focal taxon, lemurs, cannot be contaminations because no experiments were carried out with Madagascan primates in the involved laboratories. Also, the known and predicted distribution of the six lemur species (Mittermeier et al. 2008; Brown and Yoder 2015; Kamilar and Tecot 2016) agrees well with our sampling localities. Crowned lemur (*Eulemurcoronatus* (Gray)) and Sanford’s brown lemur (*E.sanfordi* Archbold) found in *H.fissicollis* and *Nanosagaboides* samples, occur in extreme north of Madagascar including the Amber Mountains. Brown lemur (*E.fulvus* (Geoffroy)), red-bellied lemur (*E.rubriventer* (Geoffroy)), eastern lesser bamboo lemur (*Hapalemurgriseus* (Link)), and diademed sifaka (*Propithecusdiadema* Bennett) have wider ranges throughout much of the eastern Madagascar lowland and mountain rain forests. The later species is classified as Critically Endangered by the International Union for the Conservation of Nature.

Our results demonstrated that Madagascan dung beetles can be considered a promising indirect tool for monitoring lemurs. They can easily be collected by standardized pitfall traps baited with ready available human feces. They can also be used in other regions, but more research is needed to assess the species-specificity of trophic relationships between beetles and mammals, as well as to evaluate the primers that are most suitable for the target mammal taxa.

With the development of amplicon metagenomics methods, they can be used for more complex analyses in the future. For example, as opposed to lemurs and other mammals, dung beetles are represented in natural history collections in large numbers from numerous localities, and they have been accumulated during decades. Thus, the rich dung beetle collections putatively contain information about occurrence of dung producer species in a particular area, sometimes not existing as a natural habitat any longer.

## ﻿Conclusions

1. Although the DNA in the gut of dung beetles is highly degraded, even minor mammal dung producers can be reliably identified by amplicon metagenomics methods.
2. Due to high sensitivity of amplicon methods in comparison to shotgun, the issue of contamination should be specifically considered, but in case of qualitative rather than quantitative studies, it does not pose a problem.
3. At present, cattle and especially humans can be the main food producers for some Madagascar dung beetle taxa.
4. Despite the fact that lemur reads may be a small percent of total mammalian reads, they allow to reliably identify lemur species that served as a food source for beetles. The 16S rDNA gene fragment used in the present study showed high specificity being rather small sized.
5. Dung beetles can be a promising indirect tool for monitoring lemurs in Madagascar.

## ﻿Additional information

### Author ORCIDs

Andrey V. Frolov https://orcid.org/0000-0002-6724-6828

Lilia A. Akhmetova https://orcid.org/0000-0002-2151-1278

Maria S. Vishnevskaya https://orcid.org/0000-0002-2634-2610

Bogdan A. Kiriukhin https://orcid.org/0000-0001-8840-2976

Olivier Montreuil https://orcid.org/0000-0003-2682-7594

Fernando Lopes https://orcid.org/0000-0002-1246-2777

Sergei I. Tarasov https://orcid.org/0000-0001-5237-2330

### Data availability

The data presented in the study are deposited in the NCBI Sequence Read Archive (SRA) database, accession number PRJNA958125.
